# Balanced Dipole Effects on Interfacial Engineering for Polymer/TiO_2_ Array Hybrid Solar Cells

**DOI:** 10.1186/s11671-017-1867-5

**Published:** 2017-02-03

**Authors:** Fan Wu, Yanyan Zhu, Xunheng Ye, Xiaoyi Li, Yanhua Tong, Jiaxing Xu

**Affiliations:** 1 School of Science and Key Lab of Optoelectronic Materials and Devices, Huzhou University, Huzhou, 313000 People’s Republic of China; 2 Department of Material Chemistry, Huzhou University, Huzhou, 313000 People’s Republic of China

**Keywords:** TiO_2_, Array, Hybrid solar cells, Interfacial engineering

## Abstract

The polymer/TiO_2_ array heterojunction interfacial characteristics can be tailored by balanced dipole effects through integration of TiO_2_-quantum dots (QDs) and N719 at heterojunction interface, resulting in the tunable photovoltaic performance. The changes of *V*_oc_ with interfacial engineering originate from the shift of the conduction band (*E*_c_) edge in the TiO_2_ nanorod by the interfacial dipole with different directions (directed away or toward the TiO_2_ nanorod). The *J*_sc_ improvement originates from the enhanced charge separation efficiency with an improved electronic coupling property and better charge transfer property. The balanced dipole effects caused by TiO_2_-QDs and N719 modification on the device *V*_oc_ are confirmed by the changed built-in voltage *V*_bi_ and reverse saturation current density *J*_s_.

**Electronic supplementary material:**

The online version of this article (doi:10.1186/s11671-017-1867-5) contains supplementary material, which is available to authorized users.

## Background

TiO_2_ is mainly used in photocatalytic and photoelectrode for photocurrent because of its nontoxicity, high electron mobility, and high chemical and thermal stability [1, 2]. Hybrid solar cells (HSCs) based on conjugated conducting polymers (donor) and TiO_2_ nanocrystals (acceptor) have received extensive attention, as they have the potential to offer low-cost, mechanically flexible, and up-scalable alternatives to conventional photovoltaics [3, 4]. A promising photovoltaic device structure for HSC consisting of a direct and ordered path, instead of disordered three-dimensional networks of interconnected nanoparticles for electron transport to the collecting electrode, has been proposed [5, 6]. Single-crystalline rutile TiO_2_ nanorod arrays (NRAs) are hydrothermally grown directly on fluorine-doped tin oxide (FTO) substrates as acceptors to dissociate excitons and collect electrons in a HSC, which demonstrates an enhanced power conversion efficiency compared with that of the dense TiO_2_ film-based device [7, 8]. However, in general, the polymer/pristine TiO_2_-NRA solar cells perform poorly, wherein most of the open-circuit voltage (*V*_oc_) is 0.30–0.44 V and the short-circuit current (*J*_sc_) is between 0.28–2.20 mA/cm^2^ [7–10]. It was demonstrated that the interfaces between the polymer and the nanocrystals play a crucial role in determining the photovoltaic performance. The relatively poor performance of the polymer/pristine TiO_2_-NRA solar cells can be partly attributed to the undesirable interfacial properties between the polymer and TiO_2_-NRAs [11, 12].

Optimization of the polymer/nanocrystal interface can enhance the charge separation efficiency and reduce the charge recombination and is an important issue for efficient HSC devices [13]. Therefore, to improve device performance, various studies have been performed on modified TiO_2_-NRA surfaces. For example, TiO_2_-NRA modified with an organic molecule (i.e., D149) has improved the *J*_sc_ to 3.93 mA/cm^2^ and *V*_oc_ to 0.60 V due to the improved compatibility of the interface morphology [11]; inorganic modification of TiO_2_-NRA, such as with crystalline CdS-quantum dots (QDs), normally results in an increase in *J*_sc_ to 1.51 mA/cm^2^ and *V*_oc_ of 0.45 V [7]; and modification with crystalline CdSe-QDs normally results in an increase in *J*_sc_ to 1.15 mA/cm^2^ and *V*_oc_ of 0.62 V [14]. It is obvious that both the organic and inorganic modifications differentially affect the polymer/TiO_2_-NRA devices’ performance. At present, few studies on interfacial engineering of combinations of the organic and inorganic material in the polymer/TiO_2_-NRA HSCs have been reported. Zhang et al. studied the composite interfacial modification in the P3HT/TiO_2_-NRA interface using inorganic (CdSe) and organic (N719 dye, pyridine) materials as modifiers [15]. At present, there are some limitations to improve the device performance by the method of monomodification, which leads to the moderate improvement in device efficiency. In their results, the performance of composite interfacial modification was superior to that of modifications based on a monolayer. Obviously, engineering the heterojunction interface using organic and inorganic materials simultaneously in polymer/TiO_2_-NRA HSCs is a method for further improving the photovoltaic performance.

**Fig. 1:**
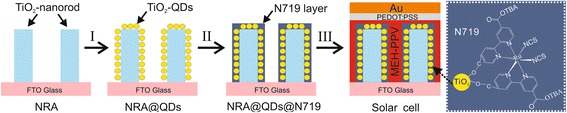
Schematic illustration for the interfacial engineering in a polymer/TiO_2_-NRA solar cell by a combination of TiO_2_-QDs and organic molecules N719 and the architecture of solar cells

This work aims at the heterojunction interface of polymer/TiO_2_-NRA HSCs, two functional materials of TiO_2_-QDs and N719 dyes are constructed at the interface of polymer/TiO_2_-NRA with certain principles as depicted in Fig. 1, which generates the synergistic effects on device performance. Results showed that the efficiency in our polymer/TiO_2_-NRA solar cells can be improved nearly fourfold by engineering the heterojunction interface. Moreover, the photovoltaic performance can be tailored through different amounts of TiO_2_-QDs and N719 at heterojunction interface, resulting in the tunable photovoltaic performance.

## Methods

### Synthesis of TiO_2_-NRA

TiO_2_-NRA was hydrothermally grown on FTO-coated glass (14 Ω/sq, 400 nm FTO thickness, Nippon Sheet Glass Co.) according to the reported procedure [16]. Deionized water (30 mL) was mixed with 30 mL of concentrated hydrochloric acid (35%) to reach a total volume of 60 mL in a Teflon-lined stainless steel autoclave (100 mL volume). The mixture was stirred in ambient conditions for 5 min, the cleaned FTO substrate was put upside down in the Teflon liner, and 1 mL of titanium (IV) isopropoxide was added. After 10 min of ultrasonic solving, the autoclave was sealed and autoclaving was conducted at 180 °C for 2 h in an electric oven to produce TiO_2_-NRA.

### Synthesis of TiO_2_-NRA@TiO_2_-QDs

The TiO_2_-NRA substrate was removed, rinsed extensively with deionized water, and dried under airflow. Subsequently, the TiO_2_-NRA substrate was put upside down in the Teflon liner and added 0.1 M titanium isopropoxide ethanol solution. The sealed autoclave was heated to 200 °C in an electric oven for another 4 h to produce TiO_2_-CSA. Once it cooled, the substrate was removed and dried under airflow after carefully rinsing it with anhydrous alcohol several times.

### Synthesis of TiO_2_-NRA@TiO_2_-QDs@N719

The dried TiO_2_-NRA@TiO_2_-QD substrate was immersed in ethanol solution of N719 (5 × 10^−6^ M) in an autoclave and heated to 80 °C for 8 h in an electric oven. After the autoclave was cooled to room temperature, the substrate was removed and rinsed with alcohol several times to remove the excess dye, providing the sample TiO_2_-NRA@TiO_2_-QDs@N719.

### Device Fabrication

The procedure used for fabrication of solar cells was similar to that described in previous works [17, 18]. Poly[2-methoxy-5-(2'-ethylhexyloxy)-p-phenylene vinylene] (MEH-PPV) (average Mn = 40000 − 70000, Aldrich) and poly(3,4-ethylene dioxythiophene):poly(styrene-sulfonate) (PEDOT:PSS) (Clevios P HC V4, H. C. Starck) were commercially obtained. The MEH-PPV layer was deposited on the top of the array by spin-coating (1500 rpm, 40 s) the MEH-PPV solution in chlorobenzene (10 mg/mL) under ambient conditions. Active layer deposition was followed by annealing at 150 °C under N_2_ atmosphere for 10 min. Subsequently, a PEDOT:PSS film was spin coated (2000 rpm, 60 s) over the polymer layer. After the deposition of PEDOT:PSS, the sample was sequentially heated for 10 min at 100 °C in a N2 glove box. Finally, a gold electrode (100 nm) was evaporated through a shadow mask to form an overlapped area of 3 mm × 3 mm between the indium tin oxide (ITO) and Au, which was defined as the effective device area.

### Characterizations and Measurements

Scanning electron microscopy (SEM) measurements of nanostructures were performed with field-emission scanning electron microscopy (FE-SEM, Hitachi S-4700). Transmission electron microscopy (TEM) and high-resolution TEM (HRTEM) studies were performed on a JEOL-2010 microscope under an acceleration voltage of 200 kV. The room temperature photoluminescence (PL) properties were measured in ambient conditions. PL measurements were made with a Hitachi F-7000 spectrofluorophotometer. The steady-state *J*−*V* curves were measured with AM 1.5 illumination under ambient conditions using a 94023A Oriel Sol3A solar simulator (Newport Stratford, Inc.) with a 450 W xenon lamp as the light source. Incident photon-to-current efficiency (IPCE) spectra of the solar cells were measured by using a QE/IPCE measurement kit (Zolix Instruments Co., Ltd.) in the spectral range of 300 − 900 nm.

## Results and Discussion

**Fig. 2:**
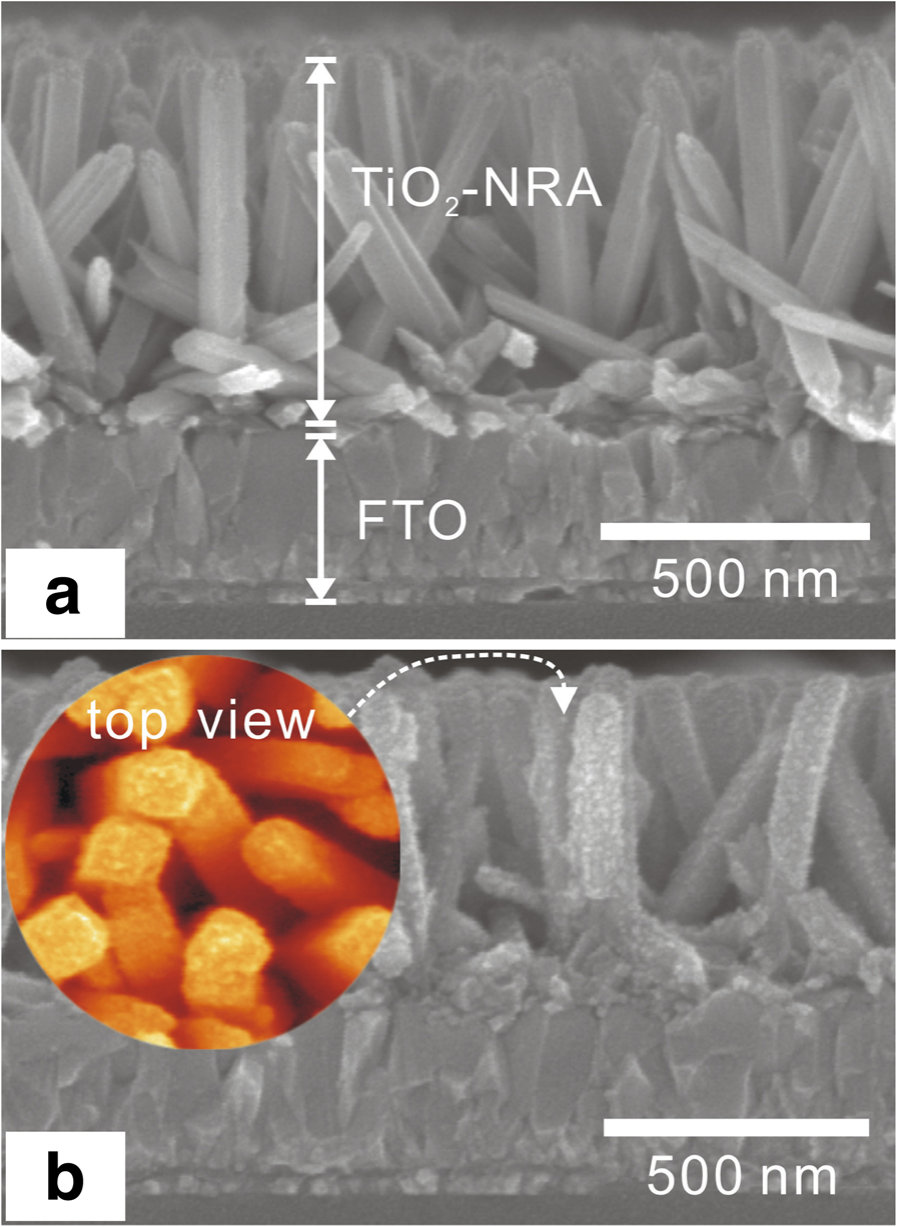
**a** and **b** are side views of the SEM images of TiO_2_-NRA@TiO_2_-QDs on the FTO substrate. The *inset* in (**b**) with pseudocolor is the top view of the TiO_2_-NRA@TiO_2_-QDs structure

**Fig. 3:**
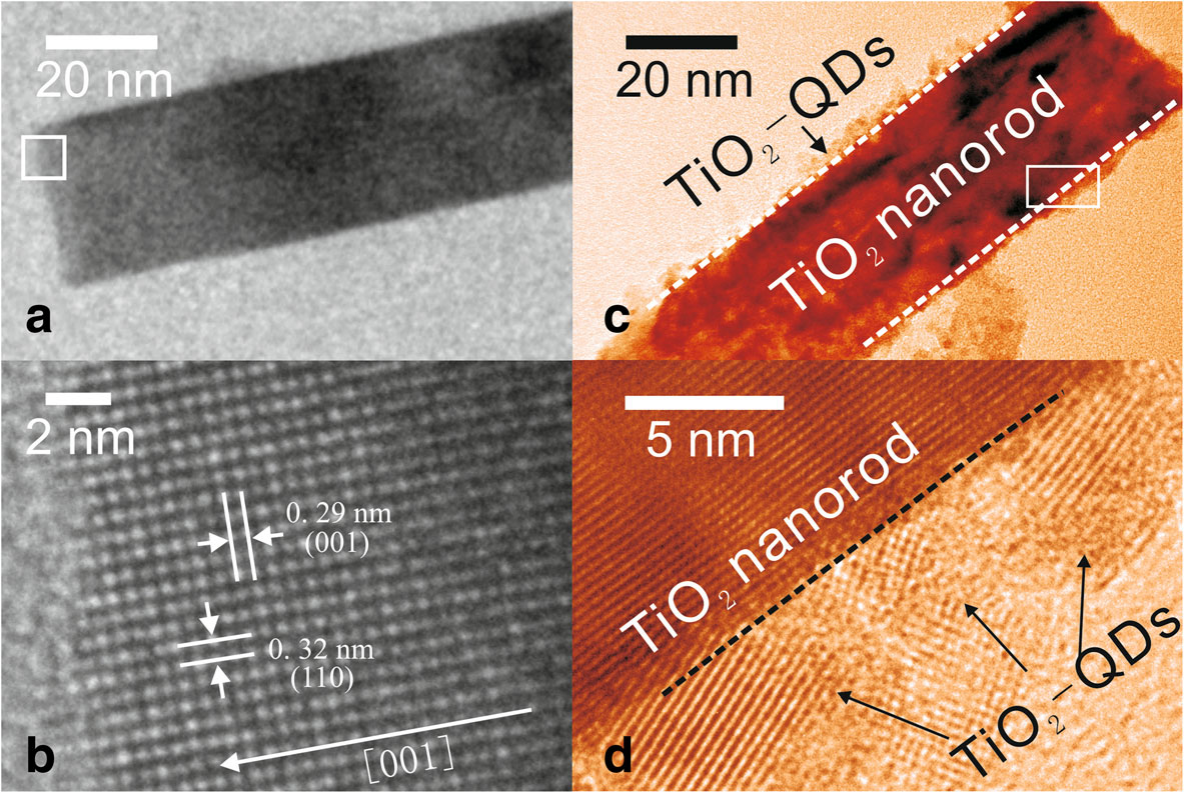
TEM (**a**, **c**) and HRTEM (**b**, **d**) images of TiO_2_-NRA (**a**, **b**) and TiO_2_-NRA@TiO_2_-QDs. The images of TiO_2_-NRA@TiO_2_-QDs (**c**, **d**) were processed with pseudocolor to distinguish them from TiO_2_-NRA (**a**, **b**). The HRTEM image (**d**) was taken from the white frame on the corresponding TEM images (**c**)

Figure 2a shows a typical side view of the SEM image of an as-synthesized TiO_2_-NRA. The rods stand almost perpendicular to the substrate, have a similar diameter in the 40 to 50 nm range, and are about 500-nm long. The TiO_2_ nanorods in the array are quite smooth surfaces. The SEM image of the TiO_2_-NRA after growth of TiO_2_-QDs (TiO_2_-NRA@TiO_2_-QDs) is presented in Fig. 2b. Compared to bare TiO_2_-NRA, obvious rough grains eventually spanning the entire nanorod can be observed in the side view and top view. The XRD pattern of the NRA@QD sample (Additional file 1: Figure S1) was only indexed to TiO_2_, which confirms that the rough grains are TiO_2_. Figure 3 shows a typical TEM image of a sample of TiO_2_-NRA and TiO_2_-NRA@TiO_2_-QDs. The TiO_2_ nanorod was single crystalline with quite a smooth surface (Fig. 3a). The lattice fringes with interplanar spacings of 0.29 and 0.32 nm match the crystal planes (001) and (110) of rutile TiO_2_, respectively (Fig. 3b) [16]. Figure 3c shows a typical TEM image of a single rod from the TiO_2_-NRA@TiO_2_-QDs. The coarse surface clearly shows that the TiO_2_ nanorod is covered by a TiO_2_-QD layer. Figure 3d is a HRTEM image of the rectangular area in Fig. 3c. The shell contains differently oriented TiO_2_-QDs with 3 − 5 nm grain sizes. The TiO_2_-NRA@TiO_2_-QD structures after bonding with (Bu_4_N)_2_(Ru)(dcbpyH)_2_(NCS)_2_ (called N719) organic molecules were characterized by the absorption spectra and the FT-IR (Additional file 1: Figure S2, in the Supplementary data), which suggest that N719 molecules are chemically grafted onto the TiO_2_ surface.

**Fig. 4:**
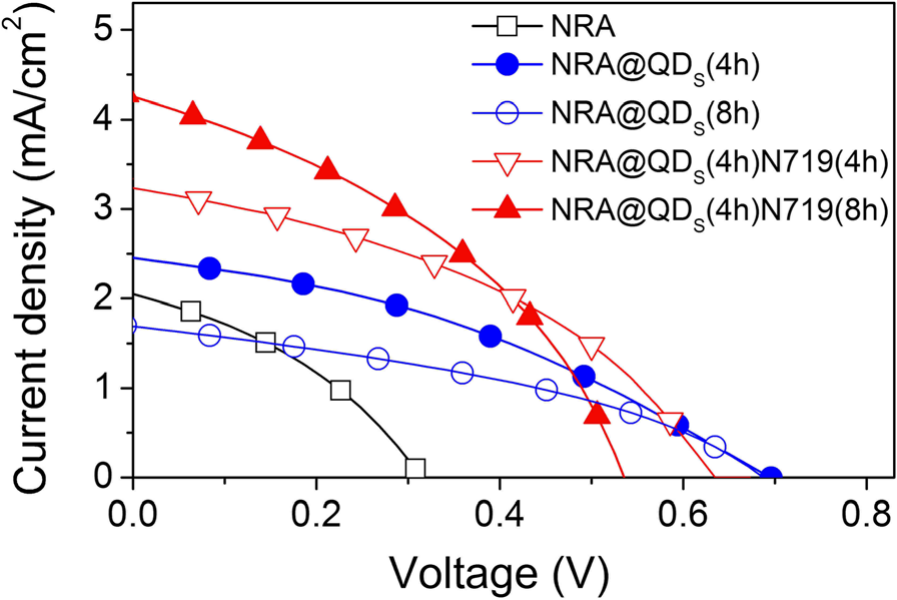
*J*−*V* curves of HSCs under the AM 1.5 illumination of 100 mWcm^−2^

HSCs based on TiO_2_-NRA, TiO_2_-NRA@TiO_2_-QDs, and TiO_2_-NRA@TiO_2_-QDs@N719 with the conjugated polymer MEH-PPV were fabricated, the architecture of the device is shown in Fig. 1. The illuminated *J*−*V* curves (Fig. 4) were measured under the AM 1.5 illumination of 100 mW/cm^2^ and the photovoltaic parameters were extracted in the Table 1. The MEH-PPV/TiO_2_-NRA device exhibits a rather low *V*_oc_ (0.314 V), *J*_sc_ (2.048 mA/cm^2^), and efficiency (0.236%) [7–12]. In contrast, after the 4-h growth of the TiO_2_-QDs on TiO_2_-NRA to form the NRA@QD structure, the *V*_oc_ are significantly improved, accompanying little improvement in *J*_sc_. Further increasing the growth time of TiO_2_-QDs will lead to a very slight increase in *V*_oc_, but the *J*_sc_ decreased because the decreased amount of polymer infiltrated into nanorod interspaces [17, 18]. After modifying the TiO_2_-NRA@TiO_2_-QDs (4 h) with N719 by an 4-h solvothermal reaction, a higher *J*_sc_ of 3.233 mA/cm^2^ was obtained, which is 2–3-fold higher than that of the MEH-PPV/pristine TiO_2_-NRA counterpart device; however, the *V*_oc_ decreased slightly. The power conversion efficiency was enhanced from 0.236 to 0.911%. We also took the N719 reaction time of 8 h to modify the TiO_2_-NRA@TiO_2_-QD sample. It was found that the *V*_oc_ was further decreased, but the *J*_sc_ (4.222 mA/cm^2^) was further improved over the sample with the 4-h reaction time. The devices also showed the good stability (Additional file 1: Figure S3, in the Supplementary data). These results suggest that *V*_oc_ and *J*_sc_ in polymer/TiO_2_-NRA solar cells can be tuned by engineering their heterojunction interface with the integration of inorganic and organic materials. The mechanism in detail of above phenomenon will be discussed as follows.

**Table 1:** Photovoltaic parameters of solar cells under the AM 1.5 illumination of 100 mWcm^−2^

	*V*_oc_ (V)	*J*_sc_ (mA/cm^2^)	FF (%)	*η* (%)
NRA	0.314	2.048	36.740	0.236
NRA@QDs (4 h)	0.695	2.450	36.218	0.616
NRA@QDs (8 h)	0.705	1.680	37.308	0.441
NRA@QDs (4 h)@N719 (4 h)	0.630	3.233	40.980	0.834
NRA@QDs (4 h)@N719 (8 h)	0.540	4.222	39.956	0.911

**Fig. 5:**
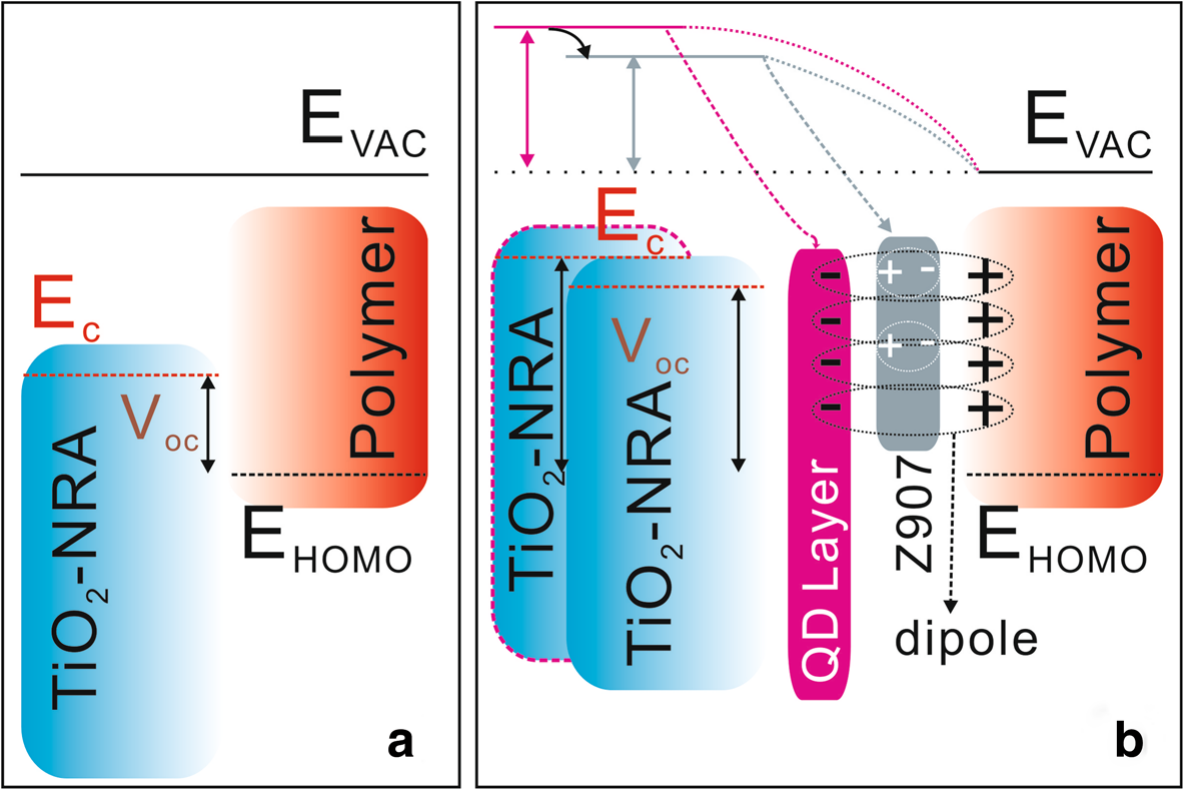
Polymer/TiO_2_-NRA band diagram (**a**) and the band structure changes subjected to interfacial engineering (**b**). *E*_vac_, *E*_c_, and *E*_HOMO_ indicate vacuum, conduction band of TiO_2_, and the highest occupied molecular orbital of the polymer energy level, respectively

It is well known that *V*_oc_ in the polymer/inorganic solar cells is mainly determined by the energy levels of the *E*_c_ edge in the inorganic material and highest occupied molecular orbital (*E*_HOMO_) band in the polymer (Fig. 5a) [6, 19]. The larger *V*_oc_ in the device with TiO_2_-NRA@TiO_2_-QDs than in the device with pristine TiO_2_-NRA has been demonstrated from the generation of interfacial dipoles in QD shell/polymer interfaces [17, 18]. The interfacial dipole generation can be considered as the formation of weakly bound pairs of electrons and holes with separations of a few nanometers by Coulombic attraction [20]. These interfacial dipoles commonly arise due to the trapping of electrons at surface states in TiO_2_-QD shell with a negative charge at the metal oxide surface and positive charge at the polymer (Fig. 5b) [19]. The *E*_c_ of the TiO_2_ nanorod can be changed by *eδE* with the presence of interfacial dipoles as is similar to the interfacial modification with dipole molecules in P3HT/TiO_2_ [21] and P3HT/ZnO solar cells [22], in which *δE* is the change of the surface potential and can be calculated from Poisson’s equation [23],

1 $$ \delta E= N\mu \cos \theta /{\varepsilon}_{\mathrm{r}}{\varepsilon}_0 $$

where *N* is the dipole concentration, *μ* the dipole moment, *θ* the angle the dipole makes to the TiO_2_ nanorod surface normal, *ε*_r_ the dielectric constant of TiO_2_, and *ε*_0_ the permittivity of free space. If dipoles are directed away from the TiO_2_ nanorod, cos*θ* > 0 and leading to *δE* > 0; if dipoles are directed toward the TiO_2_ nanorod, cos*θ* < 0, leading to *δE* < 0. Therefore, the magnitude of *E*_c_ shifting correlates with the dipole concentration and direction in the shell/polymer interface. With the presence of dipoles directed away from the TiO_2_ in the shell/polymer interface (i.e., cos*θ* > 0) due to the TiO_2_-QD shell (Fig. 8b), the *E*_c_ of the TiO_2_ nanorod core will be shifted toward the local vacuum level of the polymer due to the *δE* > 0 (Fig. 5b).

The obtained *V*_oc_ of 0.54 and 0.63 V for the MEH-PPV/TiO_2_-NRA@TiO_2_-QDs&N719-based device, however, are somewhat lower than the value of 0.69 V for the MEH-PPV/TiO_2_-NRA@TiO_2_-QD-based counterpart device. This results from the modification of the ZnO surface by N719, stemming from the dissociative adsorption of the carboxylic acid group to form a carboxylate bond, in which the positive proton charge on the surface and the negative charge on the carboxylic group together form an interfacial dipole [21, 22]. A theoretical calculation has demonstrated the direction of the dipoles generated by the adsorbed N719 molecules on the oxide surface with the monodentate anchoring mode directed to the oxide surface (i.e., cos*θ* < 0) (Fig. 5b) [22]. In this case, the dipole concentration generated by the modification with N719 will change the *E*_c_ of the TiO_2_ nanorod with *δE* < 0 based on eq. (1). That means the *V*_oc_ will be reduced by shifting the band edge potential of TiO_2_ closer to the polymer *E*_vac_ [20, 24]. The *V*_oc_ in the MEH-PPV/TiO_2_-NRA@TiO_2_-QDs&N719 device with 8 h of N719 bonding time was further confirmed in this conclusion (Fig. 2 and Table 1). The dipole concentration in the sample MEH-PPV/TiO_2_-NRA@TiO_2_-QDs&N719 with 4 h of N719 bonding time should be lower than the counterpart with 8 h. The magnitude of suppressed band edge shifting of TiO_2_ (i.e., *δE* < 0) should be smaller than the 8-h sample based on eq. (1), which causes the *V*_oc_ in the 8-h device MEH-PPV/TiO_2_-NRA@TiO_2_-QDs&N719 to be lower than MEH-PPV/TiO_2_-NRA@TiO_2_-QD&N719 with 4 h. Therefore, there is a balance of dipole effects (i.e., positive or negative of *δE* and its magnitude) by QD layer and N719 modification on device *V*_oc_.

**Fig. 6:**
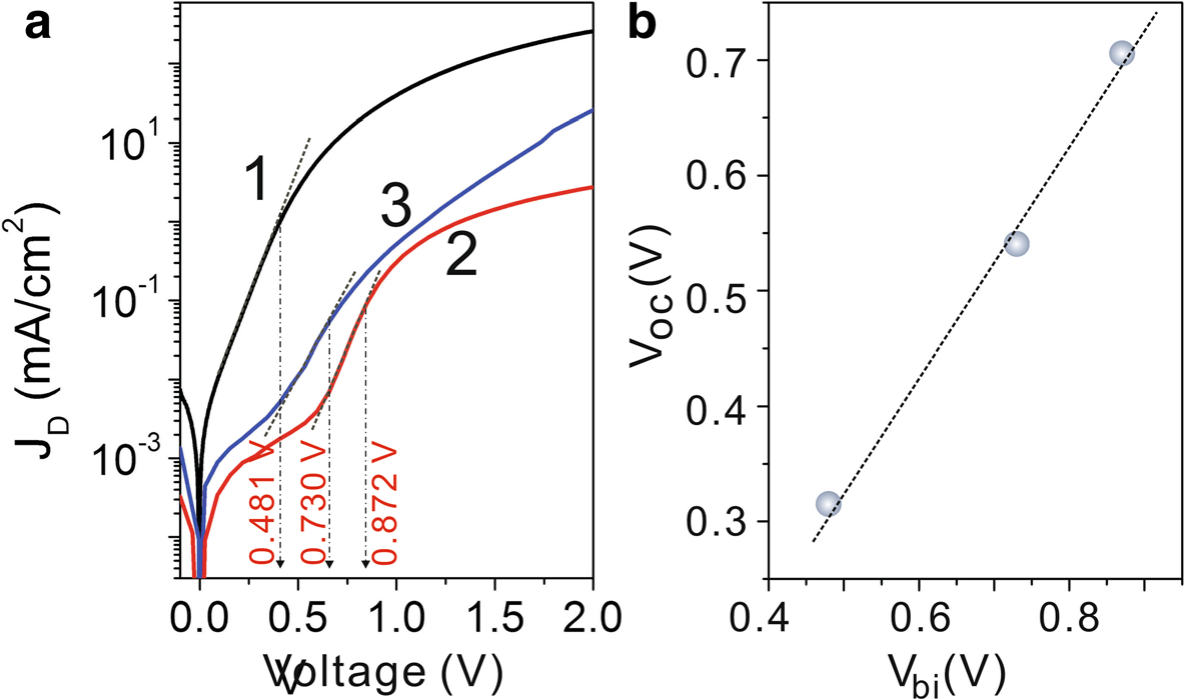
**a** Semilogarithmic plots of dark *J*_D_−*V* characteristics of devices based on TiO_2_-NRA (*1*), TiO_2_-NRA@TiO_2_-QDs (*2*), and TiO_2_-NRA@TiO_2_-QDs@N719 (*3*). The *red dash lines* indicate *V*_bi_ values. **b** Dependence of *V*_oc_ on *V*_bi_ in devices

**Fig. 7:**
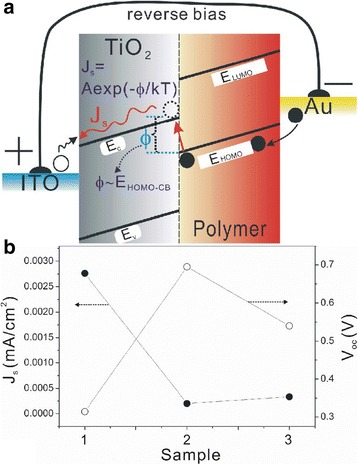
**a** Illustration of the reverse bias current *J*_s_ and energetic barrier *ϕ* in polymer/TiO_2_ HSCs. **b** Dependences of *V*_oc_ and *J*_S_, in TiO_2_-NRA- (*1*), TiO_2_-NRA@TiO_2_-QD- (*2*), and TiO_2_-NRA@TiO_2_-QDs@N719-based (*3*) solar cells. *E*_c_ and *E*_v_ indicate conduction and valence band of TiO_2_, respectively; *E*_LUMO_ and *E*_HOMO_ indicate the highest occupied molecular orbital and lowest unoccupied molecular orbital of the polymer energy level, respectively

In addition, the interfacial dipoles were confirmed by the changed built-in voltage *V*_bi_ (Fig. 6) and reverse current *J*_s_ (Fig. 7). The *V*_bi_ related to built-in electric field (*E*_bi_) which originates the work-function difference between the ITO and Au electrodes could be observed at the point where the dark *J*−*V* curve begins to follow quadratic behavior [25]. The interfacial dipoles (directed toward the polymer) could induce an extra polar electric field to enhance *E*_bi_, which is confirmed by the enhanced *V*_bi_ in Fig. 6a [22, 23]. Moreover, the *E*_c_ edge shifts in the TiO_2_ nanorod due to the number of dipole formations also agree with the changes of the reverse saturation current density *J*_s_ in the devices, which aroused our much interest. It has been demonstrated that there is often an interface activation energy barrier *Φ*_B_ at the heterojunction, which is usually explained as a result of the energy level bending by the vacuum level misalignments at the heterojunction, and could be affected by formation of interface charge transfer state or dipoles [26, 27]; the *Φ*_B_ can be evaluated from the dark reverse saturation current in dark *J*−*V* characteristic by [40.41]

2 $$ {J}_{\mathrm{s}}= A \exp \left(-\frac{\varPhi_B}{nkT}\right) $$

where *A* is a coefficient with a value in the vicinity of 1000 A/cm^2^ for the reverse bias current generation, and *k* and *T* are the Boltzmann constant and temperature, respectively.

**Fig. 8:**
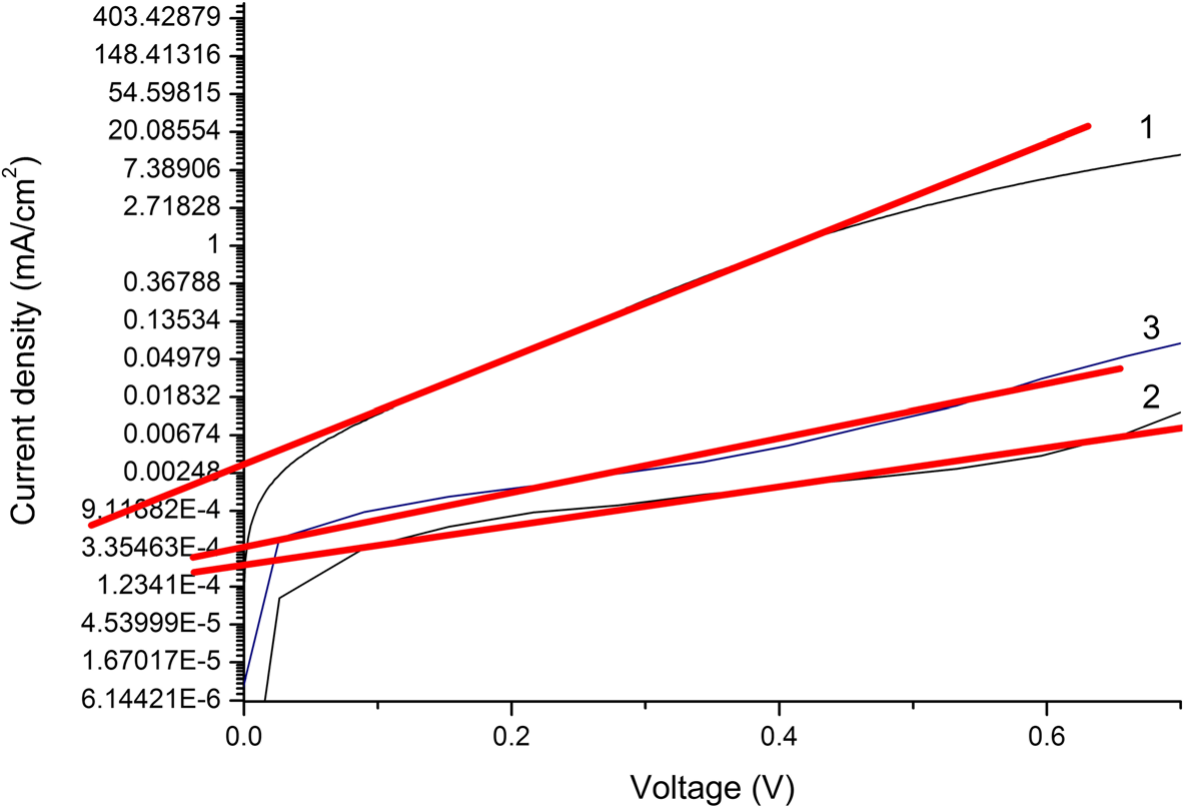
ln*J*_d_−*V* curves of solar cells measured in the dark (TiO_2_-NRA- ﻿(1), TiO_2_-NRA@TiO_2_-QD- (2), and TiO_2_-NRA@TiO_2_-QDs@N719-based (3))

The current density−voltage of solar cells in the dark can be described by the following modified ideal diode equation [28]:

3 $$ {J}_{\mathrm{d}}={J}_{\mathrm{s}}\left( \exp \left(\frac{q\left( V-{J}_{\mathrm{d}}{R}_{\mathrm{s}}\right)}{nkT}\right)-1\right) $$

where *J*_s_ is reverse saturation current density, *q* elementary charge, *V* applied voltage, *R*_s_ device series resistance, *n* diode ideality factor, *k* Boltzmann’s constant, and *T* temperature. We note that *V* > > *J*_d_ *R*_s_ for our devices, since *J*_d_ generally less than 0.01 A/cm^2^, and *R*_s_ is generally less than 200 Ω/cm^2^. Neglecting the *J*_d_ *R*_s_ term in eq. (1), for *V* ≥ *nkT*/*q*, we can get the following relation:

4 $$ \ln {J}_{\mathrm{d}}\approx \ln {J}_{\mathrm{s}}+\frac{q}{nkT} V $$

Equation (4) indicates that a plot of ln*J*_d_ versus *V* should yield a straight line. Therefore, *J*_s_ and *n* can be extracted from the ln*J*_d_−*V* curves in the linear region, which *q*/*nkT* and ln*J*_s_ corresponds to the slope and *y*-intercept, respectively (Fig. 8). Therefore, we extracted the approximate value of dark reverse saturation current *J*﻿_s_ from the dark *J*−*V* curve based on eq. 4 [29].

Based on eq. 2, we calculated the interface energy barrier *Φ*_B_ values of all devices (Fig.7b). The energy barrier *φ* for this process is correlated, but not necessarily just equal, to the difference between the *E*_c_ and *E*_HOMO_ (*E*_c_−*E*_Homo_ in Fig. 7a) due to the complicated interfacial dynamic processes [28, 30]. If there was a shift of the *E*_c_ edge in the TiO_2_ nanorod, it would affect the *E*_c_−*E*_Homo_ (i.e., *φ*), and thereby influence the *J*_s_, based on eq. 2. The changes of device *J*_s_ and *V*_oc_ with interfacial engineering are depicted in Fig. 7b. It is observed that *J*_s_ decreased from 2.76 × 10^−3^ mA/cm^2^ in the MEH-PPV/TiO_2_-NRA device to 2.03 × 10^−4^ mA/cm^2^ in the MEH-PPV/TiO_2_-NRA@TiO_2_-QD device; therefore, the energy barrier *φ* (or *E*_c_−*E*_Homo_) increased based on eq. 2, which agrees with the expectation on the up-shift of the *E*_c_ edge in the TiO_2_ nanorod after the growth of the QD layer in Fig. 5b. Additionally, the little increase of *J*_s_ (3.35 × 10^−4^ mA/cm^2^) after the engineering of N719 agrees with the small downshift of the *E*_c_ edge in the TiO_2_ nanorod (i.e., *φ*) due to the adsorbed N719 molecules on the TiO_2_-QD surface with monodentate anchoring mode directed to the TiO_2_ surface in Fig. 5b.

**Fig. 9:**
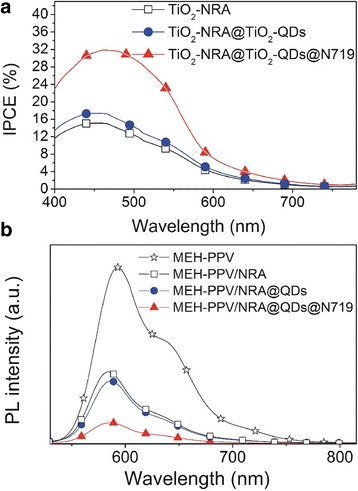
**a** IPCE spectra of solar cells based on TiO_2_-NRA, TiO_2_-NRA@TiO_2_-QD, and TiO_2_-NRA@TiO_2_-QD@N719 samples. **b** Room temperature PL spectra of the MEH-PPV and its composites with TiO_2_-NRA, TiO_2_-NRA@TiO_2_-QD, and TiO_2_-NRA@TiO_2_-QD@N719 samples

The improved *J*_sc_ in device performance after the interface modification was studied by IPCE and PL spectra (Fig. 9). The slightly enhanced IPCE (or *J*_sc_) in the MEH-PPV/TiO_2_-NRA@TiO_2_-QD device resulted from the small increase of the interfacial area due to the formation of the coarse shell for exciton dissociation in comparison to the smooth surface of the original TiO_2_ nanorods. However, the largely improved *J*_sc_ by modification of the TiO_2_-NRA@TiO_2_-QDs with N719 originates from the enhanced charge separation efficiency [22]. The amphiphilic dye improved the interface contact between the polymer and TiO_2_-NRA@TiO_2_-QDs, which improves the electronic coupling property for charge transfer. These explanations agree with the PL quenching results (Fig. 9b). Obviously, the PL intensity of the MEH-PPV/TiO_2_-NRA composite decreases significantly as compared with the intensity of pristine MEH-PPV, indicating that the PL emission of MEH-PPV can be quenched by TiO_2_ nanorods resulting from the electron transfer from MEH-PPV to TiO_2_ [31]. After engineering the TiO_2_-NRA surface with TiO_2_-QDs and TiO_2_-QDs@N719, the degree of PL quenching become more obviously, especially in the MEH-PPV/TiO_2_-NRA@TiO_2_-QDs@N719 sample. The PL quenching and IPCE (or *J*_sc_) follow similar trends indicating that the increased photocurrent upon the MEH-PPV/TiO_2_-NRA@TiO_2_-QDs@N719 indeed originates from the better charge separation and transfer at the heterojunction interface.

## Conclusions

The heterojunction interfacial engineering in polymer/TiO_2_ nanorod array (NRA) hybrid solar cells was performed in two steps: first, we grew TiO_2_-quantum dots (QDs) on a TiO_2_-NRA surface to form the TiO_2_-NRA@TiO_2_-QD structure. Next, the TiO_2_-NRA@TiO_2_-QD structure was further bonded with organic molecules (N719) on its surfaces to form the TiO_2_-NRA@TiO_2_-QDs@N719 composite array through the solvothermal method. By controlling the interfacial engineering for polymer/TiO_2_-NRA solar cells through the integration of TiO_2_-QDs and N719 molecules, the *V*_oc_ and *J*_sc_ in polymer/TiO_2_-NRA@TiO_2_-QDs@N719 solar cells can be tuned, improving the device efficiency nearly four times compared with that of pristine TiO_2_-NRA-based solar cells. The tunable device performance is resulted from the balanced interfacial dipoles, which is confirmed by the changed built-in voltage *V*_bi_ and reverse current *J*_s_. These results therefore provide information crucial to the optimization of interface in HSCs.

## Additional file

Additional file 1: Supplementary Data. Figure S1. XRD of TiO_2_-NRA and TiO_2_-NRA@TiO_2_-QDs on the FTO substrate. Figure S2. (a) UV-vis absorption spectra of TiO_2_-NRA (□), TiO_2_-NRA@TiO_2_-QDs (○), and TiO_2_-NRA@TiO_2_-QDs@N719 (△). The inset in (a) is the absorption spectra of N719 in the ethanol solution; (b) FT-IR spectra of TiO_2_-NRA@TiO_2_-QDs (1), TiO_2_-NRA@TiO_2_-QDs@N719 (2), N719 (3). Figure S3. The *J*−*V* performance of fresh device and measured after 60 days. (DOC 4431 kb)
