# Supplementary material for: Balanced Dipole Effects on Interfacial Engineering for Polymer/TiO2 Array Hybrid Solar Cells
Source: Nanoscale Res Lett. 2017 Feb 3;12:85. doi: 10.1186/s11671-017-1867-5 (PMC5291770; doi:10.1186/s11671-017-1867-5)
Supplement: Additional file 1: — Supplementary Data. Figure S1. XRD of TiO2-NRA and TiO2-NRA@TiO2-QDs on the FTO substrate. Figure S2. (a) UV-vis absorption spectra of TiO2-NRA (□), TiO2-NRA@TiO2-QDs (○), and TiO2-NRA@TiO2-QDs@N719 (△). The inset in (a) is the absorption spectra of N719 in the ethanol solution; (b) FT-IR spectra of TiO2-NRA@TiO2-QDs (1), TiO2-NRA@TiO2-QDs@N719 (2), N719 (3). Figure S3. The J−V performance of fresh device and measured after 60 days. (DOC 4431 kb) [file 11671_2017_1867_MOESM1_ESM.doc]

**Supplementary Data**

**Balanced Dipole Effects on Interfacial Engineering for** **Polymer/TiO_2_ Array Hybrid Solar Cells**

Fan Wu,*^a^ Yanyan Zhu,^a^  Xunheng Ye,^a^ Xiaoyi Li,^a^ Yanhua Tong,^b^  Jiaxing Xu ^a^

^a^ School of Science and Key Lab of Optoelectronic Materials and Devices, Huzhou University, Huzhou, 313000, People’s Republic of China;

^b^ Department of Material Chemistry, Huzhou University, Huzhou, 313000, People’s Republic of China

*Corresponding author. Tel: 0086-572-2321593; Email address:wufanjay@126.com


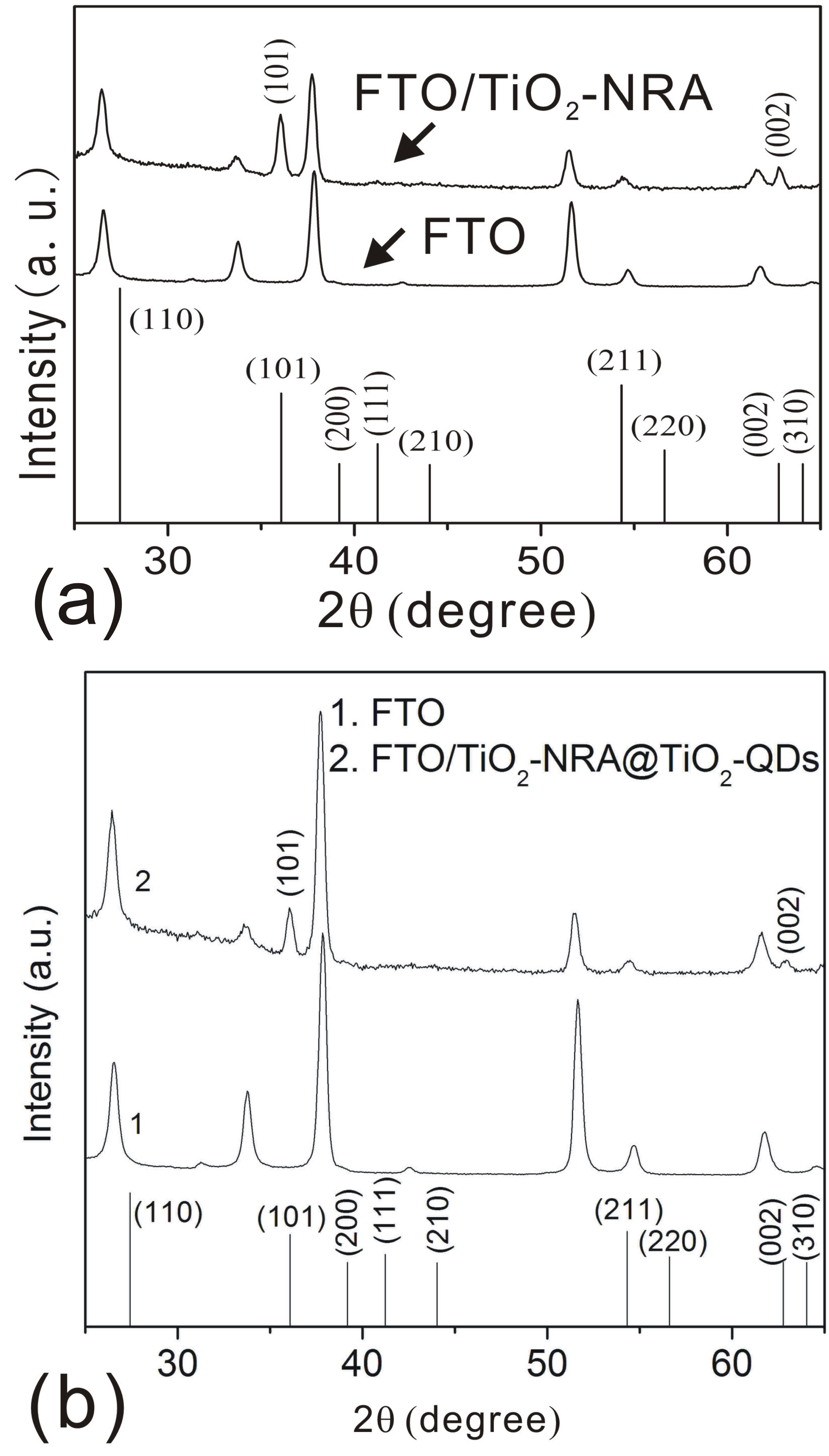


**Figure S1.** XRD of TiO_2_-NRA , and TiO_2_-NRA@TiO_2_-QDs on the FTO substrate.

**
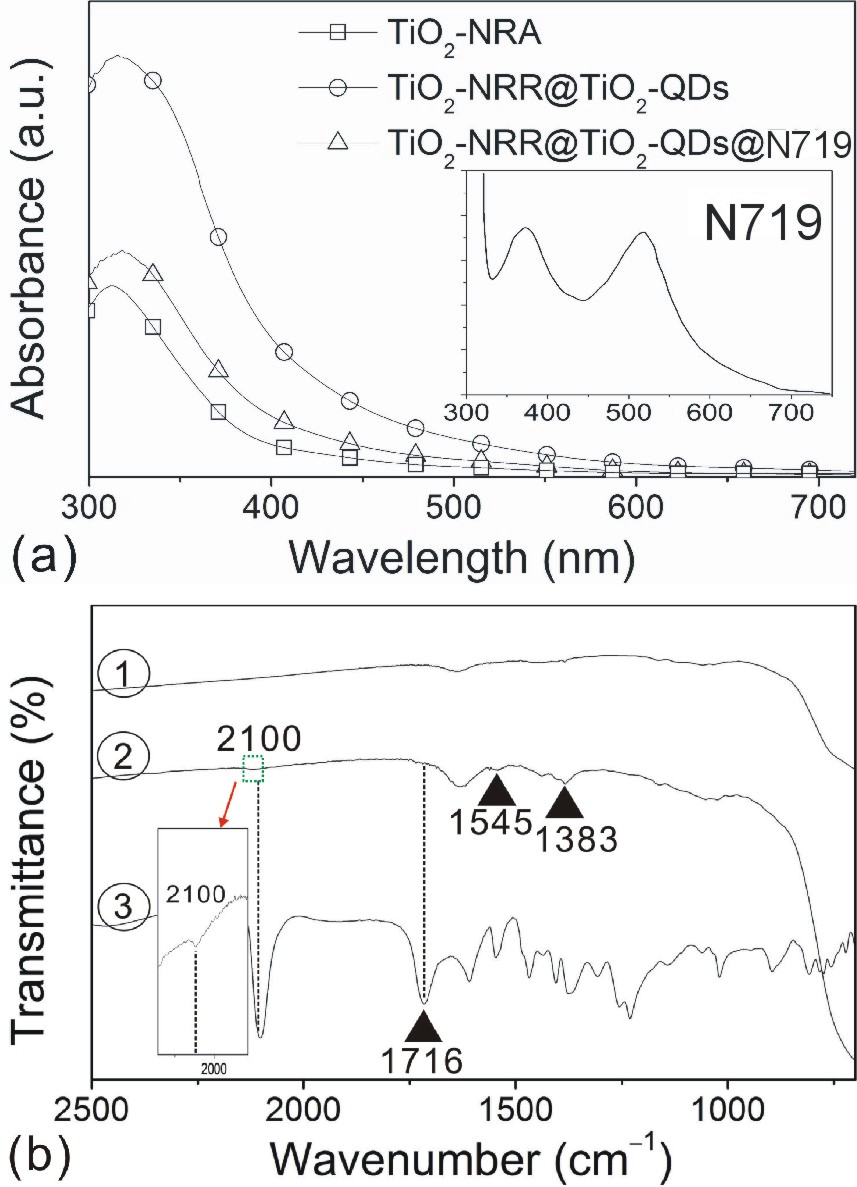
**

**Figure S2**. (a) UV-vis absorption spectra of TiO_2_-NRA (□), TiO_2_-NRA@TiO_2_-QDs (⭘), and TiO_2_-NRA@TiO_2_-QDs@N719 (△). The inset in (a) is the absorption spectra of N719 in the ethanol solution; (b) FT-IR spectra of TiO_2_-NRA@TiO_2_-QDs (1), TiO_2_-NRA@TiO_2_-QDs@N719 (2), N719 (3).

The absorption spectra (Fig. S2a) of the TiO_2_-NRA and TiO_2_-NRA@TiO_2_-QDs samples on glass substrates exhibit absorption onset at 315 nm (3.94 eV), corresponding to the band gap absorption of rutileTiO_2_. The absorption spectra of N719 (insert to Fig. S2a) show two broad bands at 386 and 527 nm, corresponding to charge-transfer transitions of the metal-to-ligand. When the N719 molecules are bonded onto the surface of the TiO_2_-NRA@TiO_2_-QDs film, a new absorption band from 300−340 nm and weak absorption from 380−600 emerge compared with the TiO_2_-NRA and TiO_2_-NRA@TiO_2_-QDs samples, which is obviously originated from the absorption of the N719 organic molecules. Fig. S2b shows the FT-IR spectra of the TiO_2_-NRA@TiO_2_-QDs, N719, and TiO_2_-NRA@TiO_2_-QDs@N719 samples. It exhibits the asymmetric (ν_as_, at 1545 cm^-1^) and symmetric (ν_s_, at 1383 cm^-1^) stretching bands of the carboxylate anion (COO^-^) complexed with surface TiO_2_ centers. The band at 2100 cm^-1^ due to the thiocyanato group (NCS) emerged in both TiO_2_-NRA@TiO_2_-QDs@N719 films. Furthermore, the separation Δν_a-s_ (= 162 cm^-1^) in the TiO_2_-NRA@TiO_2_-QDs@N719 sample is much higher than that in ionic salt zinc acetate (~94 cm^-1^), revealing a monodentate mode for N719 molecules adsorbed on the TiO_2_-QD surface. The FT-IR results suggest that each N719 molecule is chemically grafted onto the TiO_2_ surface with two carboxylic acid groups.

**
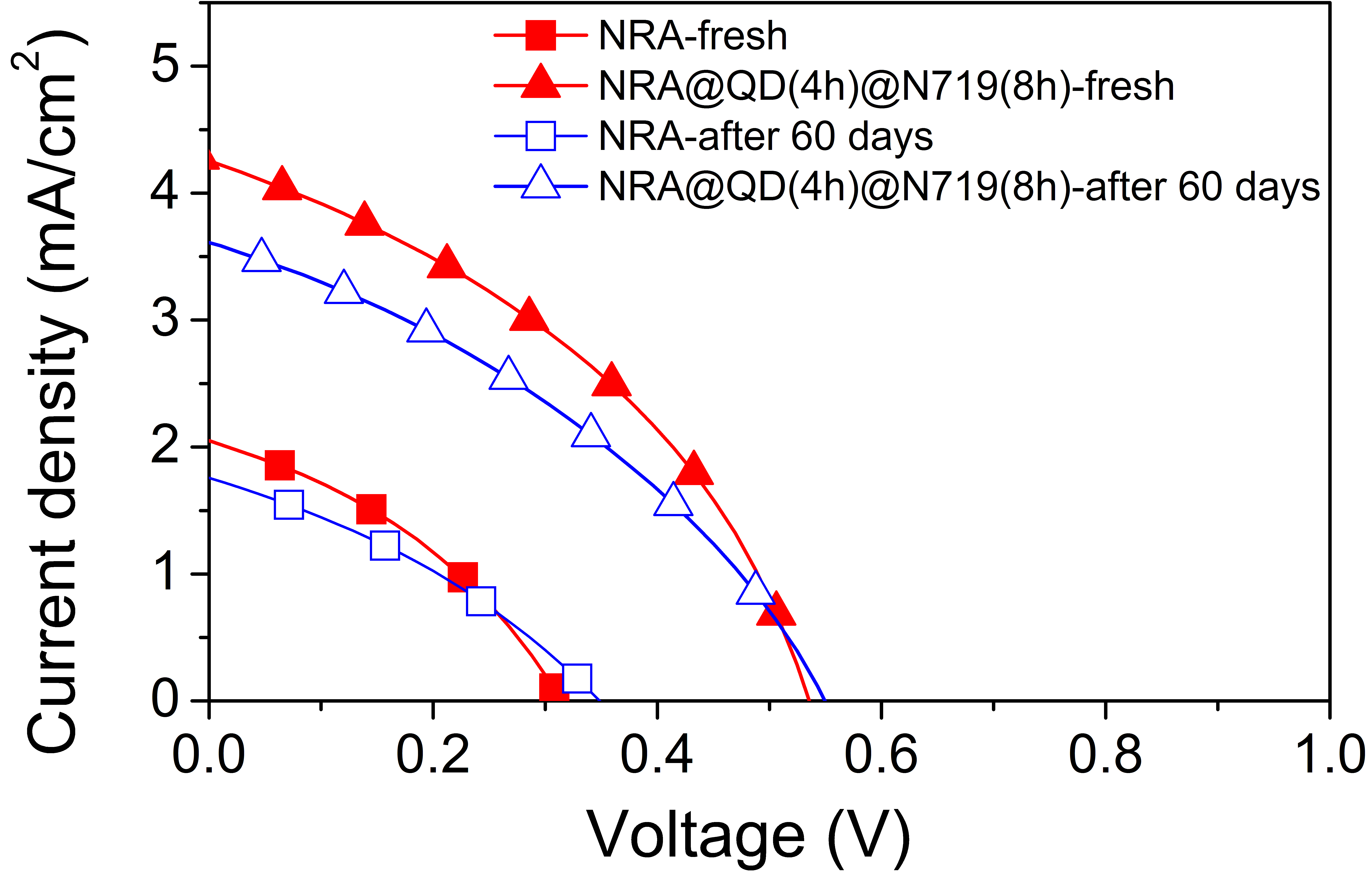
**

**Figure S3**. The *J−V* performance of fresh device and measured after 60 days.

We measured the *J−V* performance of unmodified and modified solar cells just finished and after 60 days, respectively. These devices were stored in air ambiance. Results showed that the *V*_oc_ and FF in unmodified and modified solar cells were all basically unchanged, only the *J*_sc_ was a little decreased. The TiO_2_-NRA based hybrid solar cells showed the good stability.
